# Book Reviews

**Published:** 1818-06

**Authors:** 


### CRITICAL ANALYSIS
### *OF RECENT PUBLICATIONS,*
IN THE
### DIFFERENT BRANCHES OF PHYSIC, SURGERY, &c.

*The Dublin Hospital Reports and Communications in Medicine and Surgery.* Vol. I. 8vo. Hodges and M'Arthur, Dublin; Cox and Son, London.

REGISTERS are always important, if, before the public receive them, they undergo an ordeal which may serve as a *viaticum.* It gives us much pleasure, therefore, to announce such an undertaking from a sister metropolis, which, besides its extensive population, possesses hospitals proportioned to the poverty of the inhabitants, medical schools, and an university. The plan we shall transcribe from the Preface.

"About two years ago, some professional gentlemen, attached to extensive hospitals in this city, formed the design of publishing annually Medical and Surgical Reports of the chief diseases falling under their observation, together with such incidental notices of facts and doctrines in relation to pathology, as might tend to the improvement of medical science.

"The volume which is now offered to the public is divided into two parts. The first part comprehends hospital reports, or the results of experience in some distinct classes of disease on an extended scale. The second part comprehends miscellaneous cases, illustrative both of practice and pathology, in medical and surgical diseases.

"It is proposed, if this volume be favourably received by the pro fession, to prosecute the design by annual publications of a similar kind. The work being strictly eclectic, each author is responsible for his own communications exclusively.

"Physicians and surgeons are invited to communicate digested results of their observations, on epidemic or local diseases; and to address such papers to the care of the Publishers in Dublin or London."

The first paper contains,—

*A Medical Report of the Hardwicke Fever Hospital for the Year, ending on the 31st March,* 1817. By JOHN CHEYNE, M.D. F.R.S.E. and M.R.I.A. Professor of the Practice of Physic in the School of Surgery in Ireland, one of the Physicians to the House of Industry, &c.

This paper, as might be expected, abounds with many valuable remarks. Among the first, is the discipline of the house. The additional instructions to the nurses, drawn up by the author, we conceive might form a model for every similar establishment. Our limits will not admit more than the following, which, from experience, we have often wished to offer in some form to our readers.

"When, during great derangement of mind, a patient insists upon leaving his bed, the nurse must endeavour to calm him; or, if that should fail, she may speak with authority; but she is not, on any account, to use forcible restraint. She must wrap his legs in a blanket, put on his bed-gown, and permit him to sit on his bed, or even to go to the fire, till the violence of his derangement shall abate. When indulged in this manner, he will, in general, soon return to his bed of his own accord. Patients, in fever, have perished in consequence of a struggle with their attendants, who, from a mistaken sense of duty, have endeavoured forcibly to detain them in bed, when they persisted in their efforts to leave it; whereas, had they been humoured, no injury would have arisen, provided they had been sufficiently covered.*

In addition to the above, we would remark that the

"* Shortly after I took charge of the hospital, in several instances, the maniacal paroxysm of fever, with determination to the brain, was aggravated by coercion, and hence I was led to give the above directions. The following excellent passage will be found in Grant's chapter on the Synochus Putris. 'As soon as the delirium comes on, the pain sudsides, or at least the patient does not complain of pain, or seem to feel any; but replies, in a hurried manner, when asked how he does, that he is very well; according to the observation of a French physician, "Quand le malade repond, je me porte bien, se seul mot suffit, il n'est pas plus a lui." In all these cases, the patient endeavours to get out of bed, to sit wishes of patients under fever should often be indulged when they appear even absurd. Nothing is so common in the very early dawnings of convalescence, as to find one anxious to leave his bed. If not permitted, the anxiety, if it does not protract his recovery, will render him extremely troublesome to those about him, and encrease that fretfulness which is often remarked to accompany this state of the system. If permitted, he soon discovers his own incapacity, probably before he is dressed, and is glad to return to bed, where he now remains satisfied till his physician permits him to leave it. The same may be said of diet: if anxious for it too early, he is satiated with a single mouthful. In a more advanced stage of convalescence, a much greater attention to discipline is necessary, and may be imposed without danger.

During the year 1816, the fever seems to have taken universally an inflammatory form, and blood-letting produced almost immediate good effects, if early attended to. We need therefore only mark the exceptions.

"It ought not (says Dr. Cheyne,) to be concealed, that blood-letting in two instances, in which it was performed too late in the dis-

---

up, or even to walk about from one room to another; but, unhappily, the attendants are solicitous to confine him to bed, and to load him with bed clothes; nay, he is frequently kept struggling for two or three days together, with two strong people lying upon him continually. Now, to prevent all this misery, I know no method equal to what is here recommended, namely, let the patient have his clothes put on, and be placed in an easy chair; let his head be shaven, washed with vinegar, and covered with a linen cap. When he is tired of the erect posture, let him lie along on a couch, or upon the bed, with his head high. Let his diet be cooling, and his body kept open by clysters, repeated occasionally. Let this method be persisted in till his delirium goes off; or till the pulse subsides, and he seems exhausted; then, perhaps, he will begin to doze, or slumber on his chair, which will do him no harm. When the tongue is moist, the body open, the pulse soft, and the patient seems sinking, then, and not till then, let the head be covered with a blister; give him camphor julep, with spiritus mindereri and diaphoretic antimony. After these operations, if he is inclined to go into bed, let him lie down; and, if he should remain quiet, or fall asleep, or even if a sweat should come on, let him remain in bed. But if, notwithstanding, the violent delirium should return, let him be taken up and treated as formerly. By this method, I have recovered a great number of people when I was suffered to conduct them; and I do believe some have perished by an opposite treatment that might have been saved."

case, seemed rather to accelerate the fatal termination: these cases shall be specified. It is also worthy of remark, that, in two or three cases in which venesection was performed during the exacerbatio critica, the salutary effort of the constitution was interrupted, and the fever went on for several days longer. Hence, if a rigor or sweat shall take place before the apothecary comes to execute my orders, it is my instruction that the bleeding be postponed. When a rigor took place, I directed the patient to be supplied freely with tepid diluents, contrary to the opinion of one or two of the most experienced nurses, who affirmed that 'drinks given in the time of the cool generally damped it;' but in this opinion I found they were mere theorists. A very moderate exhibition of cordials was sometimes permitted, when the struggle was violent and long continued, and the patient exhausted.

"Of the patients who had been treated in a different manner, or who had not been subjects of medical practice, but had lingered in their own miserable dwellings, under a load of filthy bed-clothes, deprived of pure air, and without having had any attention paid to their bowels for the first ten or twelve days of fever—many such objects were admitted into the hospital in the course of the summer, labouring under great debility, with a brown dry tongue, tumid hypochondria, small quick pulse, petechiæ, and delirium tending to coma. Even in these cases, I had reason to think that the disease often commenced with inflammatory symptoms. It happened on several occasions that we had to admit five or six persons from the same house, part of whom laboured under a fever with typhoid debility, while the more recent cases clearly belonged to the species of fever which I have described at length. It was observable, that such fevers as apparently had become typhoid from neglect or mismanagement, often regained their likeness to the prevailing mode of the disease, before the patients had been many days in the hospital. It is an admirable remark of Grant's, that synochus non putris becomes putrid and nervous when early evacuations are neglected. The same author somewhere remarks, that a new mode of treatment will turn an old fever into a new one. Were we to crowd our wards, shut our ventilators, give the patients a superfluity of bed-clothes, and begin early to treat the disease as typhus was treated a few years ago, namely, by bark, wine, and opium, there can be little doubt but that typhoid fevers would be the consequence.—*See Sutton's Tract on Fevers, and Sydenham Sched. Monitoria, sect.* 45."

The following passage is not less confirmatory of Dr. Jackson's remarks; and is, indeed, given almost in his words, though probably the author might not be aware to whom we are principally indebted for these important cautions.

"No man can form an estimate of the value of a fever hospital, who has not had an opportunity of witnessing the number of cases in which simply the accommodation and regimen of the house produce a favourable change in the complexion of the disease in a single night. In very many instances, the ablution of great part of the body, change of linen, the common purgatives of the hospital, the pure air of the ward, and the supply of cold diluting drinks, were productive of a crisis in the course of the first night. While, without these advantages, the disease, if we were to judge from the neglected cases, would have continued to the end of the third week, and then, perhaps, have terminated fatally; all the while the patient's attendants, or the inmates of the same house, being exposed to contagion. Because Haygarth has not made an ostentatious display of his services, the public seems to have forgotten its obligations to him.

"Next in importance to blood-letting were cathartics. Indeed it would be impossible to settle the respective claims of these remedies, which co-operate in producing the same beneficial effect. The bowels were kept soluble by moderate doses of the mildest purgatives in all who were previously blooded. It would now be idle to recommend purgatives in fever, a practice which has become universal. By the origin of that practice in Edinburgh, compensation has been made for the fatal errors which were founded on the doctrines of fever so long taught in that city. But in the practice of physic we no sooner correct one error than we fall into another: in the administration of purgatives, the bridle is often more wanted than the spurs. Much yet requires to be done, before we can consider the rules for administering purgatives and blood-letting in the different varieties of fever, and in their different stages, as sufficiently exact. We are not to prescribe blood-letting or purgatives because the disease is continued fever, nor even because it is a certain kind of fever, but because these remedies are adapted to the case under treatment. 'Fortunate medebitur qui remedii exhibendi occasiones sagax captat, quive repertæ indicationi potius quam specificæ cuidam remediorum virtuti confidit.' *Stoll.* In many cases of fever, an extravagant use of drastic purgatives, in the outset of the disease, has been highly injurious; the bowels being thereby over-excited, have refused obedience to purgatives of a moderate strength, and a disposition to tympany has taken place early, which perhaps has ended in involuntary stools. In the description of fever in question, I found mild purgatives so efficacious that I never ventured to order drastics; nor, with the exception of the hospital pills, which, by an established rule, are given to every patient who is received after the visiting hour, did I find it necessary to give a mercurial in one case of twenty."

We are forced to admit the charge against the Edinburgh School for many of the calamities which have occurred from the premature attempt at classifying fevers, and treating them according to this artificial arrangement and ill-defined names. But, though Dr. Hamilton has the undoubted merit of correcting that error, yet we conceive that the error itself only existed from the influence of a school in many other respects so justly celebrated. We are therefore ready to give Dr. Hamilton the credit of reforming that school,—but, of the general practice, no further than as it was influenced by the doctrines there taught.

The following account of the autumnal progress of these fevers deserves particular notice.

"About the end of September and beginning of October, several patients died unexpectedly. They sunk rapidly; and the symptoms and dissections concurred in explaining the cause of their death.

"In these cases the distress of the patient often bore no proportion to the danger he was in; the former was very little, while the latter was extreme. The disease would proceed without violent symptoms,—nay, a patient would seem to be recovering, although without any critical discharge: he would call for full or middle diet, and for days would take his food regularly. The only circumstance in his situation which demanded attention, was, that he regained neither flesh nor strength; he expressed no desire to leave his bed. Then his pulse again became quick, and his tongue dry, and he would complain of dull pain or uneasiness in his belly, attended with soreness on pressure, and a degree of fullness in the upper part of the abdomen; the fullness was not elastic, nor hard, nor indeed was it considerable. Then came on a loose state of the bowels, and great weakness: probably at the next visit the patient was lying upon his back, with a pale and sunk countenance, and a very quick feeble pulse,—his mind without energy. Then the stools (mucus) passed from him in bed, and the urine also; perhaps a hiccup came on; next his breathing became very frequent, in which case death was at no very great distance.

"Attempts to check the diarrhœa by means of astringents or opiates, or to rouse the patient by cordials, were alike unavailing: such remedies only seemed to accelerate death.

"By examining the bodies of those who died of this fever, I at once discovered the organ in which it chiefly resided. In October, November, and December, in every dissection, the mucus membrane of the alimentary canal was found in a state which shewed the existence of previous inflammation. It was thickened, vascular, in many parts of a bright red colour, blood sometimes was effused beneath it, the veins appeared varicose, the rugæ were prominent, and between them we often discovered small white eminences like enlarged mucus follicles, with minute apertures in the centre. 'Dans un corps sain, il est difficile d'appercevoir, sans le secours de l'art, ces follicules dans le ventricule et l'intestine grele.' *Wagler*, s. ix. 49."

When dysentery was met early by bleeding, the author found the symptoms soon relieved, whilst every other remedy, whether ipecac. neutral salts, or mercury, proved ineffectual. The functions of the brain were all this time undisturbed, the whole disease being directed to the stomach, and the rest of the alimentary canal.

After the enumeration of several other particulars, Dr. Cheyne offers an historical account of the treatment of fever, from Sydenham, through the period when his doctrine and practice were lost by the influence of the Edinburgh school; during which, one of its professors (Dr. Home) was scandalised by the occasional use of the lancet. With the revival of Sydenham's practice, he very properly compliments Jackson. We must not however forget, as he observes, our obligation to Dr. Clutterbuck, nor the effect produced by the work of Dr. Beddoes, who collected, from so many sources, the dissections of cases, showing the effects of inflammation induced by fever in all the various viscera, whilst the contents of the cranium remained unaltered. Lastly, honourable mention is made of Dr. Mills, who used the lancet in the summer of 1812, in a fever in which the abdominal viscera chiefly were affected; and, though the quantity of blood taken was inconsiderable, it was generally followed with amendment—never with apparent detriment. It is scarcely necessary to add, that all evacuating remedies should be as early in the disease as possible, or that, in an advanced stage, they are often injurious. A table follows of the probable mortality under each mode of practice; after which, we have the account of several very valuable dissections, the appearances in most of which corresponded with the symptoms during life.

*Report of certain Morbid Conditions of the Abdominal Viscera in some varieties of Maniacal Disease, with the Methods of curative Treatment;* by EDWARD PERCIVAL, M.B. Cantab. & Dub. M.R.I.A. one of the Senior Physicians to the Hospitals of the House of Industry, &c. &c. &c.

Whatever relates to this important, but hitherto obscure, subject, must interest every man, particularly if engaged in the practice of medicine. In the present day, as Dr. Percival remarks, it is peculiarly interesting, as certain notions are becoming prevalent concerning the paramount efficacy of moral management for insanity. The paper contains a number of practical remarks, but we were disappointed in not finding any dissections. That all the secretions should be attended to in this as well as in every other disease, no accurate pathologist will question; and that there are cases of insanity which, from a strong predisposition to that disease, may have been excited by certain morbid conditions of the lower viscera, will readily be admitted.

The whole concludes with some cases in which mania was the effect of, or co-existent with, epilepsy. In these, Dr. P. found good effect from turpentine; but, as most of them were of long standing, we cannot wonder if he did not produce a cure.

[The above two papers are offered as reports from the establishments to which the respective physicians are attached. The rest of the volume contains miscellaneous communications from various gentlemen in medicine and surgery.]

*On the Distortion termed Varus, or Club Feet;* by A. Colles, M.D. one of the Professors of Anatomy and Surgery to the Royal College of Surgeons in Ireland, one of the Surgeons to Dr. Stevens' Hospital, &c. &c.

Dr. Colles has found great advantage from attempting the cure of this deformity at a very early period after birth; at which time, the parts are most pliable, and require much less force to restrain them, on account of the delicacy of the muscles which the patient has not hitherto been accustomed to use with any considerable exertion. The apparatus is so simple, that a general idea may be formed of it without the assistance of the plate, which of course every one will consult before he attempts the same method of cure.

"The apparatus consists of a shoe made of chamois leather doubled, and having between its two layers a sole of strong tin interposed. The tin is cut to the size of the sole of the foot, having, however, two small projections left, one opposite to the ball of the great toe, and another opposite to the outer ankle. Each of these projections has a longitudinal slit, designed to receive the shouldered end of a splint. The splints are made of tin completely covered with chamois leather, except merely the shouldered end of each, which serves as a kind of tenant to pass down through the mortice-like slit in either projection, and which should be left of such a length as to admit a hole capable of receiving a bit of very narrow tape, or strong bobbin, below the sole of the shoe. The splint for the outer side of the limb is a narrow strip of an uniform breadth and shape; that for the inner side somewhat resembles the leg on which hosiers stretch their stockings. They should each be about an inch broad, and of such length, as to reach up to that point where the leg swells out below the inner condyle of the tibia. The upper leather of the shoe is entirely open in front, being cut down through the middle, from the instep to the toe. Along the edges of this cut are four or five corresponding holes, through which the ends of a doubled lace are alternately passed crossways from the toe to the instep; and, to protect the foot from being injured by the friction of this lace, a false lining of soft leather is interposed, which is sewed underneath to one of the edges. The point of the shoe is completely open, the upper leather being cut away across the toes, so as to leave them entirely exposed. All the edges of the shoe, from heel to toe, are bound with tape or narrow ribband; and to the heel below the edging are sewed two straps of leather, of such a length as to cross once or twice on the instep.—Such is the apparatus. The mode of applying it is as follows: The foot being introduced into the shoe, the assistant presses the heel of the shoe against the heel of the foot, while the surgeon laces it as tight as is necessary to keep the foot steadily in its position, and then secures the end of the lace by passing them once or twice round the foot, and tying them on the sole. He next crosses the straps of leather sewed to the heel once or twice on the tarsus, and ties them either on the sole or on the instep. The use of the shoe is to draw closer together the anterior ends of the metatarsal bones, particularly of the great toe, which stands off to a distance from the others, while it serves at the same time gently to press the bones of the tarsus into their proper places. The straps from the heel assist also in pressing these latter bones still farther into their natural position; but the principal use of these straps is to prevent the heel from rising up out of the shoe. When the shoe is adjusted, the splints are then to be applied; and the shouldered end of each being inserted into the longitudinal slit designed to receive it, two pieces of bobbin, each about two inches long, are passed into the two small holes below the sole of the shoe; and their outer ends being knotted, their inner ends are tied together across the sole. The limb is then brought into the natural position, and the splints being adjusted to it, so as to preserve it in that position, are secured in their places by two tapes sewed to the outer splint at convenient distances. The use of the splints is sufficiently obvious. The outer splint having its lower end fastened to the sole of the shoe, must, from the distorted state of the foot, have its upper end very much turned out from the leg. It is evident, therefore, that when this splint is pressed in against the leg, it must necessarily force the outer edge of the foot upwards, so as to bring the sole more into its natural position, the splint in this case acting as a lever, and the outer ankle serving as a fulcrum. Again, the inner splint being of one single piece, and being made in the same direction as the perfect limb, it is evident that when the leg and foot of the splint are firmly secured to the leg and foot of the child, the foot must, by the pressure on the ball of the great toe, necessarily be brought into a straight line with the leg; while, at the same time, the shouldered end of the splint must keep down the inner edge of the foot, and prevent it from returning into its original distorted position. In adjusting the apparatus, great care must be taken that the upper ends of the splints do not press against the sides of the leg, so as to rub off the skin, and this will be best accomplished by bending these ends outwardly.

"The inner splint has in general a great tendency to slip backwards, and then, in the bended position of the knee, to excoriate the upper part of the leg. This may be guarded against by passing the upper tape of the outer splint in front of the tibia, then round the inner splint, and so back again to the outer side of the leg."

To prevent the apparatus from being wetted with urine, a napkin folded lengthways is wrapt spirally round the limb, from the toes to the knee. This the nurse is directed to change as often as it is moist. It serves also as a defence from the sharper points of the apparatus. The author concludes with some notice of the instruments proposed by others. Though he prefers his own, he very candidly suggests the possibility of differences in the deformity of different subjects.

*Observations on the Remittent Fever, and on the Plague which prevailed in the Island of Corfu, during* 1815 *and* 1816. By William Goodison, M.D. Assistant Surgeon to the 75th Regiment.

This paper, though short, contains many useful hints, particularly on the advantage derived from Peruvian bark in cases of intermittent, and the probability that the prejudices of the islanders against that remedy arose from its occasional failure, and from their confounding the intermittent with the remittent fever. In the last case, that remedy, if not injurious, was at least useless.

Impartiality obliges us to copy the author's opinions and arguments relative to the contagious property of the plague, especially as the compiler of the Reports remarks that "their value is very much enhanced from the observations having been made without a view to the support of any peculiar doctrines."

"With respect to the contagion, (says the author,) the following facts may not be uninteresting. A woman was taken ill within a month of her time; she was delivered in two days,—after which she only survived three. She had no symptoms or mark of plague upon her body; however I judged it prudent to send the family, consisting of six persons, including the infant, to the camp, to be placed in observation; after six days, a thrifty old lady of the family, having got herself settled, took a handful of cotton to spin; she had no sooner set to, than she was taken ill with vomiting; a young man was also infected before night, and the whole six were exterminated in a week, with five other persons in an adjoining building, who had had communication with them. I traced the contagion in two instances to pack-saddles which were infected. In one of these cases the father, who used the saddle, escaped; but his son, a boy about nine years of age, caught the infection, and died in three days. The mother, who carried the boy to the camp in her arms, a distance of six miles, left her house in perfect health, was attacked the following day, and died in thirty hours with petechiæ, carbuncle, and bubo. I think we are but ill acquainted with the limits of the operation of contagion and the laws of susceptibility. Most extraordinary and unaccountable instances occurred, where the greatest caution, excited by the greatest terror, could not shield the unfortunate victims of this horrible disease; of course the infection was conveyed, in all these instances, by contact, which, in the ever multiplying sources of danger, unexpectedly took place; yet, of 300 Albanese soldiers, employed in guarding the infected, following the track of contagion hourly, patrolling the streets at night, and, above all, guarding the expurgators in their various offices, we lost but two, a captain and his servant. Yet they could not take any precaution about their shoes and clothes. In many families in this quarter the mortality was deplorable, fifteen dying out of sixteen infected, at the lowest calculation: this was, however, owing in some measure to the remoteness of the Lazaretto, which was six miles off. With respect to the introduction of the disease, I am firmly persuaded that it was imported, although I know many believe it to be indigenous, and they have some strong reasons to support this opinion. First, they say we cannot trace out the fact of its introduction, notwithstanding the offer of a large reward. Secondly, that the district has been always remarkably unhealthy, and twice had medical officers been sent down two successive autumns, to ascertain the nature of a suspicious epidemic. Thirdly, the character of the disease varying so much: at first it appeared in the form of cynanche maligna, nor did parotids appear until some time after this period, and buboes much later, accompanied by convulsions and sudden deaths. The period of the disease also varies; at first it extended to five and seven days, latterly, three days was the mean duration. Most of the patients I saw died in forty-eight hours, and there is reason to believe that some were cut off in twelve hours.

"In answer to all these: First, the whole nearly of the village of Marathea, where it broke out, was destroyed, certainly all that could know any thing about it. Secondly, the exemption of all the other parts of the island outside the cordon, (although subject to all the circumstances assumed as the cause of the disease at Marathea, viz. bad air, the consequent generation of a contagious distemper, aggravated by poverty and every sort of privation). Add to this, that although the district had been always unhealthy, no disease, known there before, approximated to this in its proportion of mortality (*ab initio*) to convalescence. The ratio was nearly inverted, one death occurring in sixteen cases of the severest remittents, during a summer; whereas fifteen died of plague, for every one that recovered. Thirdly, I believe it always happens that plague makes its appearance in an ambiguous form, and shifts and changes about like a Proteus.

"I know not what apology to make for this long string of facts and opinions, all jumbled together, but that, if I had had more leisure, I should have been more attentive to order. I was determined, however, to dispatch them even so, and just set them down as they occurred in my note-book.

The argument, that the writer was warped by no preconcerted theory, should be allowed to have its share of weight: at the same time, it must be admitted, that a person who had formed some previous opinions of his own might have been more methodical in his remarks and in his manner of relating them. The only danger would have been lest he should have dwelt too long on some facts, and hastily passed over others. In the present instance, there is no reason to suspect either; but the candid author, by his concluding language, admits the doubtfulness of any legitimate conclusion from the facts as far as they are established. Our readers are aware that we are very shy of this same Proteus. In the elegant fable we have of him in the Odyssey, we find him a very crafty fellow, capable of almost any disguise; yet, when a prudent man was well instructed, well supported, and had patience and courage to conduct himself properly, he not only was more than a match for Proteus, but, besides detecting his real shape, learned many particulars from him which he could not have gained by any other means. We may, perhaps, take another opportunity of introducing this same Proteus to our younger readers. At present, it is enough to say, that whenever they avail themselves of the heathen deities to help them out of difficulties, they should, at the same time, reflect on the advice which Hercules gave to the waggoner.

*Account of an Epidemic Petechial Febricula;* by Edward Percival, M.B. Cantab. and Dub. M. R. I. A. one of the Senior Physicians to the Hospitals of the House of Industry, &c. &c. &c.

This is an interesting little paper. A slight fever, attended with petechiæ in its early stage, invaded a female seminary, and spread through the whole family. Whether it was contagious or merely epidemic, does not appear with certainty; for, if not confined almost to the house, there were no proofs of its existence any where but in its neighbourhood, nor of the manner in which it was introduced.

"Two of the patients, one a child only five years old, the other an adult female domestic, were sent to me in Dublin, as specimens of the disease. The woman was then in the fourth day of her febricula. The chilliness, of which she had at first complained, had entirely subsided. Her tongue was thinly coated with mucus, her eyes were somewhat dull and watery, her pulse about 86, and perfectly tranquil. She had no giddiness of the head, pain, or cough, and complained only of languor, thirst, and impaired appetite. She had numerous small petechiæ, perfectly distinct, of a dun colour, which she informed me appeared on the second day of her indisposition. The stigmata which I saw, were scattered on the neck, shoulders, and fore arm; and she told me there were similar spots on her loins and lower limbs. The child had contracted the same disorder about the same time. The petechiæ on her trunk and limbs were numerous, but more faded in their hue, than in the other case. She was lively, and occupied in her sports nearly as usual; yet her countenance obviously betrayed indisposition, her appetite was impaired, and her bowels costive.

"In each case, I directed only gentle purgatives, to be repeated when occasion required; and the children who remained at the school were treated in a similar manner. Had I been on the spot to watch the first access of the febrile symptoms, I should undoubtedly have tried the efficacy of emetics or cold affusion, in cutting short the disease *in limine*. But the first stage being passed, no further occasion appeared for active interference.

"The duration of the febricula did not, I believe, exceed nine days in any instance, and the petechial eruption lasted about five days. No sequel of cutaneous or intestinal ailment followed. Some scattered cases of the disorder occurred in the neighbourhood of the school and the village of Delgany; but not a single case, (so far as I have been able to learn) degenerated into typhus fever.

"I am quite unable to conjecture the origin, or assign any probable cause of this remarkable febricula. It may be worthy of notice, that in the preceding winter (1815) a very slight morbillous epidemic appeared in Dublin, attended with the characteristic eruptions, but paler and more scanty than usual, with little fever or coryza. Its progress through two female schools fell within my own observation; and of the patients whom I attended, *four* experienced a second attack of the disorder, in a much severer form, within the space of three months. I at first doubted the accuracy of my previous observation; but facts of a similar kind occurring to other practitioners in the city, at the same time, I was confirmed in the opinion that my patients had twice undergone the measles in the course of one winter.

"But the petechial appearances in the fever before described, bore no resemblance to the morbillous eruption. Neither roughness nor elevation of the cuticle were observed, nor even the marbled efflorescence, which often occurs in typhoid fevers. The spots were in fact precisely such stigmata as appear in the fevers commonly termed putrid or malignant, without any collateral symptom of inflammation, putrescence, or malignity.

"How far these facts will serve to throw light on the proximate cause of petechiæ, I do not venture to determine. The disease termed *hæmorrhæa petechialis,* ought to furnish further illustration of the same subject. The common theory of inflammation appears to me as little calculated to explain these diversified phenomena, as the obscure doctrine of putridity. Were I to indulge any conjectures on the subject, they would be founded on the disturbed balance of the venous and arterious systems—laxity of the subcutaneous capillary veins, and defective absorbent power of the capillary arteries."

*Some brief Notices of the Deleterious and the Medicinal Effects of Green Tea;* by the same.

In two cases,—one in the author's own practice, the other communicated by Dr. Hervey,—an over-quantity of strong green tea induced palpitation, incubus, and something bordering on asphyxia. The first was greatly relieved by opium. This suggested to Dr. Percival the idea that tea might prove a better antidote against the effect of opium than coffee or many other remedies usually at hand. The following is the only case in which he made the trial.

"About four years ago, a very delicate valetudinarian lady swallowed two ounces of the camphorated tincture of opium, mistaking it for a purgative draught. I saw her about three hours afterwards, and found her overcome by deep sleep, so as scarcely to be roused by all the efforts of her attendants. We removed her from her bed to an erect posture in her chair, and fomented her legs. With great difficulty, I prevailed upon her to swallow a cup full of strong green tea, which soon proved emetic. After some bilious discharge from her stomach, she drank freely of the tea in a more diluted state, and in a few hours recovered entirely from the effects of her inadvertence. The quantity of opium which she had swallowed in the tincture, did not probably exceed four grains, from which no permanent ill effect might have been expected: but I did not trace, in the after-course of the gouty disorder under which she then laboured, any vestige even of temporary mischief from the accident.—I mention these facts, rather as hints, than as affording any solid evidence for relying upon green tea as a remedy in cases of a more alarming nature."

Several other good practical observations are contained in the paper. In tea, as in hops, the familiar use has (as Dr. P. remarks) rather withdrawn than attracted scientific enquiry into the peculiar effects it may produce.

*Case of Melæna, with Observations on the alternate Excess of Morbid Action in the Mucous and Serous Membranes;* by J. Cheyne, M.D. &c.

This paper contains many practical remarks; but, we confess ourselves not quite satisfied with all the author's inductions. That ascites is often the mere effect of effusion from peritoneal inflammation, we cannot doubt; yet, we have often been disappointed in our attempts to cure it by purgatives of any description. When the disease has been attended with violent pain in its early stage, we have been more successful with this and other evacuations; but ascites is a formidable disease, and arises, we suspect, from various causes. The cases of melæna, though they ended fatally, are valuable, as they contain the dissection; yet, we know not how to satisfy ourselves that all this black blood is furnished by hæmorrhage from the stomach. If it be, we have still to learn why it should assume such a colour and peculiar consistence, without the power of coagulation. It seems somewhat similar to the black vomit in the yellow-fever, allowing that the latter may be diluted with other fluids.

*Of Jaundice unaccompanied with any discoverable Disease of the Liver, or Turgescence, or Obstruction, of the Biliary Ducts;* by the same.

A youth of fifteen, a native of Quebec, was admitted into Hardwick Hospital on the ninth day of a fever. He was brought from the House of Industry, in which a fever, having all the characters of typhus, had been often endemic till within the last year, more especially during the season at which this boy was admitted. When brought to the Hospital, besides the customary symptoms of fever, the skin and tunica adnata were of a deep yellow colour: he took purges and blue pills, and died early in the morning of the fourth day after his admittance.

" *Dissection.*—The stomach, intestines, and liver, were apparently free from disease. The gall-bladder contained a small quantity of light-coloured bile, and the biliary ducts were unobstructed throughout their whole extent. The spleen was rather large. The kidneys were remarkably diseased—they were much enlarged; their outer surface was marbled; and, on cutting into them, we could hardly distinguish a trace of their original structure. The ureters were rather contracted; the capacity of the bladder was diminished, its coats were thickened, and the mucus membrane was diseased. A sound was introduced without any difficulty into the bladder.

" The fluid contained in the pericardium and pleura, was yellow and viscid, but it was scarcely increased in quantity.

" There was an effusion of yellow serum between the arachnoid membrane and pia mater; the coagula in the longitudinal sinus partook of the yellow tinge. The ventricles were considerably enlarged, and contained a yellow fluid to the amount of several ounces; their inner surface was spotted in a peculiar manner; the spots bore a strong resemblance to petechiæ. These spots were seated in the lining membrane of the ventricles. The brain was remarkably firm in its consistence."

"If (continues Dr. C.) we are to retain typhus as a generic term for our most severe contagious fevers, this case may be counted a specimen of typhus icterodes. The term typhus icterodes, as applied by Sauvages and Cullen to the remittent of warm countries, carries with it a nosological impropriety; for, if so applied, a species of fever which arises from the noxious influence of the soil, would be classed with those fevers which are produced by the fomites of contagion.

"The continued fever, which generally prevails in summer, (*synochus biliosa, vel gastrica*) is not unfrequently attended with hepatic inflammation and congestion, and yellowness of the skin; but typhus, attended with jaundice, is a rare specimen of disease. In the course of the last eighteen months I have seen only two specimens of typhus icterodes in at least fifteen hundred instances of fever.

"The yellowness of the skin, in the case of Walsh, did not take place in patches or spots, similar to what is observable after ecchymosis: the urine was of a deep yellow colour, and in the stools there was a sufficiency of bile; the eyes were of an orange colour; the suffusion was general and deep, as in confirmed jaundice."

"If!" as this ingenious author observes. May we not then hope, that the word Typhus will, for a time, be banished, till it has some appropriate meaning? Has it not already done mischief enough? Has it done any good whatever? It is, however, some comfort to see it gradually vanishing, and, when it appears now, to find it always attended with some qualifying epithet, doubt, or apology.—This borders on disgrace.

*On the Cause of the Disease termed Trismus Nascentium;* by A. Colles, M.D. one of the Professors of Anatomy and Surgery to the Royal College of Surgeons in Ireland, one of the Surgeons to Dr. Stevens's Hospital, &c. &c.

In this ingenious paper, Dr. Colles produces many arguments to show, that trismus nascentium is the same as trismus traumaticus, and arises from the suppuration of the umbilical cord. He, therefore, proposes a variety of modes of treating the cord during separation, and acquaints us with the practice among the negroes of the West-India islands, in whose children this disease used to be particularly fatal. At present, it is scarcely known, and the difference is imputed to dressing the umbilical cord with spirits of turpentine, and plunging the infant daily into cold water. We would also suggest, whether a few experiments might not be made of leaving the cord untied, making the division not too early after the birth of the child, and carefully watching the event. Among the inferior animals, trismus nascentium, as far as we know, has not been observed.

*Case of Dropsy, by Conversion of Disease, from the Ski the Serous and Cellular Membrane;* by Edw. Percival, M.B. Cantab. and Dub. M.R.I.A. one of the Senior Physicians to the Hospitals of the House of Industry, &c. &c.

This is one of those numerous cases which seem to remind us of the importance of attending to the danger of inflammation, during convalescence from acute disease. The candid author is very sensible of this; but few people are aware how strongly it was first urged by Dr. Jackson, and that the consequence of his withholding a full diet, for a certain time, was an accusation, that, from motives of economy, he deprived our brave defenders of that necessary nourishment, without which convalescence was rendered more tardy, and relapses more frequent. It is some gratification that he lives to see the public undeceived in these respects, though few are aware of the source to which we owe this improved practice.

*On the Virtues of James's Powder in the Apoplectic Diathesis;* by J. Cheyne, M.D. &c.

Every practitioner must be aware of those unhappy cases in which we are perpetually forced to bleed, on account of threats of apoplexy; yet the patient becomes, by degrees, far from plethoric. Under such cases, Dr. Cheyne has found the use of Dr. James's powder, in small doses and long continued, sufficient to take off this strong determination of the blood to the head. The following is his mode of administering the remedy.

"The patient begins with a very moderate dose, not more than two grains at bed-time, and to increase the dose by half a grain every night, until some sensible effect is produced upon the stomach, bowels, or skin. Should the stomach be affected with sickness, the dose must be lessened by one grain on the following night. By the addition of a little rhubarb to it, a larger quantity of James's powder may be administered than the stomach could otherwise bear. If the skin is softened, or the bowels affected, the dose should not further be increased, but it must be repeated every night for a considerable length of time: in several instances, I have known eighteen or twenty grains taken for a considerable period without any inconvenience. Even when not productive of any sensible perspiration, it sometimes allays the heat and restlessness which so often attend irregular determinations of blood. In one or two instances, it has apparently had a soporific effect. In one instance, the patient, after a comfortable night, always awoke gently perspiring, his skin previously having been remarkably unyielding; but, in other cases, it has produced no sensible effect upon the secretions or excretions, save that of removing a sense of burning heat of the surface of the body."

No ill effects were produced by the remedies, either on the appetite or indigestion, nor did the habit of perspiration induce any greater susceptibility to rheumatic complaints.

*History of a remarkable Enlargement of the Biliary Duct;* by CHARLES H. TODD, Member of the Royal College of Surgeons in Ireland, &c.

We do not immediately recollect a case exactly similar to this. It appears as if, by some preceding inflammation, the opening of the *ductus chadedochus communis* into the duodenum had been obliterated; in consequence of which, the bile, being continually secreted, had enlarged the hepatic and common duct. These contained more than a quart of bile, forming a sac which extended behind the intestines, from the *porta* to the *sacrum*, and, in a transverse direction, covering the right kidney and part of the left. A puncture into the gall-bladder was made during life, without shortening the patient's life, as far as can be judged from the symptoms.

*On Periostitis, or Inflammation of the Periosteum;* by PHILIP CRAMPTON, M.D. F.R.S. Surgeon-general to the Army and Forces in Ireland.

This, with some surgical cases, will be considered in a future number.

---

*Practical Illustrations of the Scarlet Fever, Measles, Pulmonary Consumption, and Chronic Diseases; with Remarks on Sulphureous Water, &c.* By JOHN ARMSTRONG, M.D. 8vo. Baldwin and Co. Pp. 448.

THE abilities, industry, and accuracy, of Dr. Armstrong, are already well known to our readers, and, we trust, duly appreciated in our Journal (see vol. 37, p. 305). The subjects of the present volume are all of practical importance; and, if they do not derive the same interest as the work which came before under our inspection, it is only because they have already been so much noticed by some of the ablest writers in the profession.

Scarlet fever is divided, as by Dr. Willan, into simplex, anginosa, and maligna. We mention not this to undervalue Dr. Armstrong, because we conceive a better division could not be made; besides which, Willan's work is not in every one's hands; and luminous as it is in description, diagnosis, and prognosis, the practical part is much too guarded—as in most of his work we see too little of his own practice, and more than is necessary of other people's. In all these respects Dr. A. has the advantage: but we expected frequent reference to so celebrated a writer, as a mark of respect and gratitude for the extraordinary services of one who had laboured with so much diligence in the same field.

We have always held that scarlet fever, as well as all other fevers, should be treated according to their symptoms, and not according to their names; and that this treatment should therefore be bold and early, without waiting to ascertain the name of the disease. The diagnostic is, however, highly important in all those diseases which occur only once during life; for by such accurate knowledge only can we assure our patient of his security from each during the remainder of his life. In this respect we must still refer to the accuracy of Willan, whose diligence, so conspicuous in every part of his work, is in none more so than in scarlatina, and its discrimination from measles. The practical remarks of Dr. Armstrong are all judicious; but we confess, in the truly malignant form of the disease, which most happily is of rare occurrence, we have found ourselves unsuccessful under the boldest use of evacuants, cold or partial warm effusions, or both, or mercurials, or any other practice, however early we may have commenced our career.

At the conclusion, we meet with some very just remarks on the danger attending convalescence, from the high irritability of the subject during the restorative process which succeeds most acute diseases. To these the author very properly ascribes the dropsical symptoms which sometimes follow scarlatina, and this view of the subject leads to the prevention or cure of these sequæ læ, by withholding too great indulgence in diet, and by careful depletions, as soon as symptoms of inflammation appear.

Dr. A. urges very much, that, when scarlet fever is epidemic in any family, the throats, particularly of the younger part, though in apparent health, should be carefully examined. Some preternatural appearances about the fauces will frequently, he remarks, give us early warning of the disease, the importance of attending to which he illustrates by a very interesting case. We would urge this attention on another account: in many families we shall find the disease in one individual with all its threatening symptoms, in another so mild as to pass unnoticed, without this diligence and sagacity of the attentive practitioner. But the importance of ascertaining the fact is great, on account of the future security of the subject, as was mentioned above. The passage, to which we now refer, leads to another on which we are forced to make some observation.

"Before concluding this subject (says our author,) it ought to be noticed, that most of the older authors are for, and most of the later against, depletion in the malignant forms; so various are the records of human opinion, even on matters of vital importance. The theories of medical men are constantly changing, but diseases have always been under the same influences; as the planets revolved by the same laws, whatever conjectures were framed respecting them in the lapse of ages. The opinions of men may vary, but the operations of nature are unchangeable. The three powers which affect disease, independent of the physician, are—the cause, the circumstances under which that cause is applied, and the condition of the subject upon whom that cause and those circumstances operate. Now one and the same cause always produces similar effects, and, though the effects are modified by peculiarities of places and of patients, yet still they are reducible to varieties which observe regular laws; and, if those varieties had always been marked and separated with sufficient care, instead of having been confounded in general descriptions, what now seems a chaos in physic would have presented an harmonious arrangement. One very remarkable example might be adduced, to shew how the same cause produces different, yet ascertainable, varieties of disease, and that is the miasm arising from damp or marshy grounds: in one person it occasions an intermittent, in another a remittent, and in a third a continued fever,* according to the place where it originated, or to the habit in which it occurred; and, notwithstanding these types of fever differ among themselves, yet singly considered, they are subject to a surprising uniformity of character. Only attentively examine the effects produced by any other specific cause of febrile disease, and it will be satisfactorily discovered, that each of them admits of a similar uniformity; and indeed it is by ascertaining the nature of each of these effects, that we are principally enabled to deduce precise rules of treatment.

"How then, it may naturally be asked, has it come to pass that so much discrepancy of theory and of treatment should exist concerning febrile diseases? There is a tendency in the human mind rather to search after the abstract essences of things, than to collect, arrange, and exhibit their phenomena as presented by nature; and this tendency, it cannot be fairly denied, has operated as powerfully in medicine, as in any other department of scientific inquiry. Hence, since Hippocrates, comparatively few have minutely noted either the varieties or the stages of particular fevers, either the peculiarities which surrounded, or which existed within patients; and hence, too, the labours of innumerable practitioners have

---

* "Dr Cullen has excluded from his definition of the continuæ, fevers which arise from marsh effluvia, as if they did not occasionally assume the continued type. But in the common systems of nosology, we constantly find diseases, not arranged according to their natures, but according to the theories of the respective writers."

been unproductive of good, from an implicit reliance upon the hypothesis of some ingenious predecessor or cotemporary. One conjecture or other, relative to the scarlet fever, has exerted a manifest influence over most of those who have written on the subject; so that their respective opinions are coloured by the doctrine of the day, as rays of light are tinged by the medium through which they pass. But so far as my reading extends, all those writers who decidedly condemn depletion, either do so on the vague authority of others, or from having themselves used it partially in the first, or seen it destructively hazarded in the last stage. Indeed, a singular confusion exists both in the descriptions and in the opinions of almost all the writers who protest against venesection; for not only do they blend the symptoms of the first and the last stages together, but carry their prejudices so far, as to censure purging with the most indiscriminate zeal. It were to be wished, that they had at least paid more attention to the stages; for it is upon this point that all the difficulty, that all the discrepancy, turns, in the treatment of the malignant varieties. Let one man, called very early, pursue the depletory plan, and he will save the greater part of his patients; let another, called late, pursue the same method, and he would lose every patient. It is for want of having discriminated the stages that those authors have erred in their own practice—it is for want of having discriminated the stages, that they have unconsciously misled the public through their literary productions. The late most excellent Dr. Fothergill, who so powerfully advocated the doctrine of debility in his popular essay, changed his sentiments when his experience became matured; for a little before his death he confessed to Dr. Withering, that he had long been dissatisfied with the treatment of the scarlet fever, and that in prescribing bark, he had rather yielded to the opinion of others, than followed the suggestions of his own judgment.* Those authors were successful who used depletion early, and those failed who used it late; and thus they arrived at different conclusions respecting the same measure, because it was employed under circumstances essentially different. But we are coming fast round to the old practice, for purgative medicines, through the agency of Dr. Hamilton, are now universally employed in the scarlatina anginosa; and, if to this method that of the cold and warm affusions be added, we shall perhaps find the treatment of no modification of disease so much improved in modern times. The result is felt every where. Practitioners acknowledge, that the scarlet fever has become far less fatal since the introduction of these measures; and there is no denying the assertion, since the scarlatina anginosa is by far the most common form of the disease. But, though the malignant varieties of this affection are fortunately of

---

"* See p. 10, of An Account of the Scarlet Fever and Sore Throat; the second edition. By William Withering, M.D. F.R.S. Birmingham, 1793."

the least frequent occurrence, yet we must still expect them occasionally to return, and should be prepared to encounter them with a promptitude and decision suitable to their extreme urgency."

That "one and the same cause always produces similar effects" no one can question; yet there are many objections to the illustrations of the forms of diseases by the revolution of planets. The latter are subject to no deviation, as far as human wisdom can inform us; but in disease, as Celsus very justly remarks, "*deinde non sequitur ut quod alium non affecit aut eundem alias, id ne alteri quidam aut eidem, tempore alio noceat Cels. Profatio,*" and, as Dr. Armstrong afterwards reminds us, there are always two causes, the *causa mali*, which may be the same with all, and the original or accidental condition of the subject, which is peculiar to each. These we have no means of calculating upon: nor even on the modifications which the original cause may derive from seasons and other conditions of the atmosphere, still more remote from our observation than idiosyncracies. On this account not only Hippocrates, but Sydenham, with far greater accuracy, marked the character of fever according to the effect which the unknown constitution of the air induced; and, in this manner, had his successors continued to correct their practice by observation, we conceive that much less discrepancy would have arisen between different writers, and even in the same writers, than we now meet with. We think Dr. Fothergill, too, somewhat unfairly treated. Whoever peruses his treatise on Scarlatina with sufficient diligence, will find many cases in which he advised bleeding, for what he entitled the ulcerous sore throat, or scarlet fever. But, in his days, he found it necessary to check that immediate recourse to the lancet, which, from the advice of Sydenham, and writers of the highest antiquity, had been the practice in every kind of sore throat. Sydenham had evidently never seen any form of scarlatina but the mildest, and in which the throat, if at all, was very slightly effected. The probability is that Fothergill, as well as Sydenham, lived to see a revolution in the constitution of the atmosphere, which might render an alteration of his practice necessary. His first treatise was written before 1750, and he lived more than thirty years afterwards. Necessary as we may now find depletion in the commencement of most acute diseases, some of us may live to see the necessity of greater caution. But this apology is hardly required for Fothergill, who, in every edition of his book, describes cases which require the lancet.*

* It is worth remarking, that the disease was usually called the *putrid* sore throat, which probably led to the stimulating prac-

In the treatise on Measles we find occasional references to Dr. Willan, in passages which, on account of their importance, we shall select.

"When (says Dr. A.) pain or soreness in the chest, oppressed breathing, general anxiety, and restlessness, are absent in the eruptive stage of the measles, we shall have no occasion to bleed; and where these are present, venesection, either general or local, will almost always be necessary. But sometimes it happens, that the breathing is very hurried, the cough frequent, and the pulse much quickened, about the first coming out of the rash, and yet, if we wait a day or so, we shall find the respiration gradually improve; and no doubt Dr. Willan alludes to cases of this kind when he judiciously observes, that those, who from doubt, or from some collateral motives, are led to await the event, usually find the pulse become moderate, and the uneasy respiration terminate in twenty-four hours. In irritable children, and especially in infants, the respiration often becomes extremely anxious on an attack, even of simple fever, wholly unconnected with pectoral inflammation; and this is more particularly the case when the bowels are disordered. We must be most careful to discriminate such a state of breathing from that which commonly attends pulmonic inflammation: nor, indeed, is this difficult, because the former is seldom permanently the same, but varies so much, at different times, that the patient will seem now much oppressed, and again easy; whereas, in the latter, there are no changes of this sort in the respiration, for it is so considerably oppressed, as never to be entirely easy. Besides, the anxious breathing, which arises from irritation, is generally increased by the erect position, and that which arises from inflammation, more or less diminished: in the first, the child now and then obtains pretty tranquil slumbers, with little motion of the chest; but, in the last, the sleeps are always very disturbed, and the chest may be seen heaving up and down with an unnatural labour. When any part of the pulmonary system is inflamed in children, both the diaphragm and the abdominal muscles are generally thrown into an inordinate action; so that if the belly and breast be exposed, one cannot fail of being struck with the circumstance; and forcible movements of the last-named muscles may often be remarked about the navel. In very young children labouring under pleuritis, or any similar affection, we cannot of course obtain an account as to the feeling of pain, soreness, or uneasiness, and the like, that may exist in the chest; but the permanent oppression of the breathing, the above-mentioned state of the diaphragm and abdominal muscles, the general restlessness, and the peculiar anxiety of the countenance, may lead to a tolerably correct opinion."

On this occasion we think it necessary to remark, that we have not unfrequently met with a subject, under measles,

---

tice; but Dr. Fothergill, in all the editions we have seen, denominates it *ulcerous* throat, or throat with ulcers.

in all other respects seeming to suffer little, yet with a respiration so quick, as to resemble the panting of one who had run himself out of breath. This is so far from being a rare occurrence, that we now view it with indifference where the patient has no pain or other bad symptom. This *anhelitus celeritas*, which continues without interruption during sleep, and in every posture whilst awake, is of great importance for young practitioners to be aware of, as it can hardly fail to induce an unnecessary alarm to those who have not previously witnessed it before.

We perfectly agree with Dr. Armstrong, and some other writers, that Watson has not been fairly treated by Willan's suspicion that he mistook scarlatina for measles, but we are by no means satisfied that the disease had not a gangrenous tendency from the beginning. Though our limits will not permit us to go into all the minutiæ of this part of the volume, yet we may be permitted to remark that the disease is traced by Watson with much accuracy from its commencement, and through all its stages, to its sequel, in a manner which marks the author as an accurate philosopher, and good practical physician.

Pulmonary consumption is the next subject to which Dr. Armstrong calls our attention, and which occupies a third part of the book. Ingenious as many of the observations are, we cannot help thinking that they might have been comprized in a much shorter compass. All the moral suggestions we conceive might have been spared, not from their want of importance, but because they are tolerably well known to the physician, and even to the patient. We believe, too, that the plan of this essay is less original than the author suspects. At the close of the book we meet with an apology, that he was unacquainted with the writings of M. Bayle when his work was printed. Had he referred to our Journal, he would have found even that gentleman anticipated. Page 307 of our fifth volume, published so long ago as the first year of the present century, contains an arrangement so similar to M. Bayle's and Dr. Armstrong's, that it will not be easy to perceive the difference, excepting that the latter deems many of the diseases, to which all three writers refer, rather as disease resembling phthisis, than as phthisis in a different form. These are—chronic inflammation of the bronchia, ulceration of the trachea, chronic inflammation of the pleura, chronic and common inflammation of the lungs. The chronic inflammation of the pleura has not, indeed, been enumerated among the causes of phthisis, but rather of hydrothorax. But, if this is an addition of Dr. Armstrong's, we suspect that he underrates hæmoptoe in the same proportion. He conceives it more frequently the effect, than the cause, of phthisis, and that in nine out of ten cases, blood is spit up from the bursting of tubercles which have previously existed. There may be, he believes, some solitary cases in which the retention of coagula in the body of the lungs excites them to suppuration; but this suppuration, he conceives, is more commonly of the ordinary, than of the true phthisical, kind (page 251).

That, on the rupture of a tubercle, streaks of blood will be sometimes mixed with pus, no one can question; but surely this is very different from hæmoptoe, as described by the ablest writers. That suppuration of tubercles is a different process from the suppuration of coagula, or from the broken ends of blood-vessels, we are not disposed to question; but either of them we conceive fairly entitled to the term phthisis, if sufficiently considerable in extent to prove fatal. We are forced to suspect it can only arise from the particular sphere of Dr. Armstrong's practice, that he considers hæmoptoe so very rare a cause of this disease.

Of the curative methods proposed by the author, his attention to the skin is the most important, the most copious, and best arranged. We make no objections to the rest, excepting to the use of caustics, vesications to other parts, and other external sources of irritation. To us they appear useless, and only encrease the misery of the patient. It must, however, be acknowledged, that in these dreary cases we are forced to be doing something, and the sanguine expectations of the patient make him fancy that he finds relief from whatever is done. On this last subject, our author shows that laudable spirit of improvement which it does not become us to repress, arrived perhaps at the age described by the poet,—

———" Inventis miser abstinet ac timet uti

" Vel quod res omnes timide gelideque ministrat"—

We shall, however, transcribe the introductory part of his plan.

" By way of putting the subject of consumption into a fair train of enquiry, I would propose, that an institution should be founded in London, expressly for the purpose of investigating the nature and treatment of this disease; and, that annual reports of its progress should be laid before professional men of every country, with whom a correspondence ought to be solicited in regard to its objects. To increase astronomical knowledge, Dr. Halley engaged philosophers of different countries to watch the transit of Venus over the sun's disk; and, surely the consideration of consumption is of far more importance to the world,—a disease which yearly occasions the death of so many thousands at the most interesting or useful ages. This institution should be upon a large scale, and divided into two departments: the one for incipient, and the other for confirmed, cases of consumption. It should be erected in an airy situation, and so planned, that each patient might have an excellent bed-room and sitting-room, opening into each other upon the same floor; and these rooms should have a separate apparatus for raising or reducing their temperature to any point required; and they ought, likewise, to be furnished with double windows in cold weather. If this institution were once established, much might be suggested for its internal management, so as to make it comprise almost every thing likely to contribute to the comfort or utility of the patients; and, though it would be highly gratifying to me to have the medical superintendance of such an institution; yet, if any practitioner can accomplish its establishment, I will unreservedly communicate whatever appears in my view most fitted to give it beneficial force and effect.

"In reviewing what has been written, I find that one or two circumstances have been omitted relative to the prevention of pulmonary consumption, and I shall therefore make no apology for introducing them here. It would appear from Gibbon, the splendid narrator of the decline and fall of their empire, that the Romans, even in the reign of Augustus, had probably neither linen nor glass among their domestic and personal comforts; and, certainly, the manufacture and use of these two commodities are most manifestly acquisitions of real utility to the modern world. With respect to glass, while it fully admits the light into our rooms, it secures them from the inclemencies of the weather; and, if we could fall upon a more rational mode of heating them, our houses might be made still more effectual defences against the influences of a cold and variable climate. The present method of having the fire at the middle or corner of a room is extremely injudicious: because, if the room be of a tolerable size it only warms one part, and leaves the other comparatively cold; and, to this circumstance alone, may sometimes be traced the origin of coughs which end in consumption, not to mention other disorders of the viscera. The skin is the medium through which many external impressions are communicated to the vital organs within; and it is most extensively and intimately concerned in the phenomena of several acute and chronic disorders. But it is, especially during convalescence, from diseases which leave great weakness, that colds are apt to be caught on account of the irregular temperature of our houses; because the skin is then far more susceptible than in a state of strength, and, because the internal organs are then far less able to resist external impressions, by reason of the general debility. Under such circumstances, therefore, great care ought to be taken as to the temperature of the sitting and bed-rooms, when there is the least apprehension of a tendency to phthisis; and, indeed, this is a point which should never be neglected in persons hereditarily tainted, even when they are apparently in the best health. If some individuals of practical minds, and of personal consequence, would direct their attention to some better mode of warming our houses, their labours might be productive of great national advantage: for I do firmly believe, that consumption, in particular, would be less common, if the temperature of our houses were rendered more equable and agreeable, by the use of stoves and flues, as in some parts of the continent, or, as in the hot-houses of our own gardens; and, not only would there be much less risk within doors, in our worst seasons, from the vicissitudes of the weather, but we should always be able to go warm into a cold atmosphere, which is the best way of resisting its influence. The metropolis alone presents a little world to our consideration, the health and comfort of which might be very much increased by introducing a more effectual and cheaper method of warming houses; and, if the improvement were extended to the whole united kingdom, the benefit resulting from it would be incalculably great. The thing is quite practicable, and, by co-operation and perseverance, might be speedily carried into complete effect. In fact, as hydrogen gas can now be so easily extracted from coal, our houses might be warmed and lighted by a modification of the same apparatus; and, as small coals, which are exceedingly cheap, would answer for these purposes, an immense reduction of domestic expenditure would be the result."

Many very ingenious and useful proposals follow, which we sincerely wish may be attempted, and would gladly indulge a hope of their success.

A few essays are subjoined incidental to various parts of the foregoing work. The first is on the efficacy of balsam of copaiva in inflammation of mucous membranes. The good effect of this remedy in chronic gonorrhœa very fairly suggested to the author the probability that some advantage might be derived from it in increased secretion of the trachea or bronchial vessels. Probably, it was formerly found, that the balsams were serviceable in these kinds of pulmonary discharges, which might have led to the custom of administering them in other forms of phthisis, a practice so much condemned by the celebrated Dr Fothergill.

The next paper is on chronic diseases and sulphureous waters. Here we meet with many useful practical remarks; but, we conceive, the paper might have been shortened with advantage, by the omission of all that is said concerning the enormous crimes of gluttony and drunkenness; not only because the subject is pretty well understood, but because those vices can hardly be said to increase among us. This paper contains also a practical remark of some importance, namely, that a small quantity of blood, taken by leeches, will frequently induce a very considerable effect on the action of the heart and arteries.

In considering the virtues of sulphureous waters, the author very naturally reverts to the late plan of substituting them for mercury in many chronic diseases. We sincerely join with him in this preference; and, like him, we shall wait for further experience before we offer our opinion on the effects of these waters in phthisical complaints. We shall also await the further elucidation which Dr. Armstrong seems to promise us before we say any thing on the classing of the functions of the body into vital, mechanical, and chemical. To us, they all appear vital, because neither mechanism nor chemistry can produce any of the effects which we find from the nerves, arteries, and fluids, during life—and during life only; nor can we comprehend how their actions should be altered by disease, if we are to view these organs as merely mechanical or chemical.

The author concludes by a promise to present the public, in the course of the year, with two Essays: one on the Excitive Fever; the other on Water in the Brain. This excitive fever is that called by Cullen Synocha and Synochus, or, as Dr. A. remarks, it may comprehend almost all disorders in his order Phlegmasiæ.—Certainly Nosology is a very accommodating science!

Such are the contents of this volume. That it contains much good matter cannot be questioned; but, should a new edition be called for before the author has completed his two promised essays, we conceive enough may be spared from the present volume to leave room for them both.

---

*Account of an Epidemic Small-Pox, which occurred in Cupar in Fife, in the Spring of* 1817; *and the degree of protecting Influence which Vaccination afforded; accompanied with Practical Inferences and Observations.* By HENRY DEWAR, M.D. F.R.S.E. and Fellow of the Royal College of Physicians of Edinburgh. 8vo. Pp. 38. Longman and Co. London.

THE most remarkable circumstance in this history is the number of cases in which small-pox occurred after vaccination; and that in all, excepting one, the disease appeared to be mitigated—passing through its stages quicker, and without secondary fever. It will be enough, therefore, if we particularize the solitary case.

"David Philp, son of a master-wright, aged eleven, was vaccinated at an early age.

"On the present occasion, was taken ill on a Sunday with a complaint in his head. The eruption appeared on the following Tuesday.—On Wednesday, he was seized with a vomiting which never left him. On Tuesday and Wednesday, the interstices of the pustules were occupied with a general rash similar to meazles: this however went in. Mr. Wiseman, a medical student, who attended him, says, that the pustules were regular and thick. He continued to complain much of his head. Leeches were applied on the Thursday, and at the time he received considerable relief: afterward, however, he complained of severe pain in the abdomen and small of the back. The bowels were not constipated, but the stools were preternatural, and resembled minced meat. Petechiæ were observed on his breast before his death, which took place on Friday, the fifth day of his illness. After death, his body was black with petechiæ."

In a subsequent passage, we have the following additional remarks on the same case.

"The uniform absence of secondary fever would be pleasing to the systematic medical observer, as showing one positive law of vaccination. But to mankind in general, more intent on practical good than on theoretic consistency, it would be more satisfactory if we could say, that no case of small-pox in a person who had been vaccinated, proved fatal. It therefore becomes a matter of importance to take a fair view of the unfortunate case of David Philp. *He did not live till the eighth day*, and thus there was no opportunity of seeing whether he would have recovered at that period with equal suddenness as the others, whose previous symptoms were most severe. It must also be observed, that this was a case complicated with visceral disease, which, probably at one time, if not till his death, partook somewhat of the inflammatory character. Other symptoms of an opposite nature, viz. petechiæ, were present, so that this boy was in the same state as a person in the most rapidly destructive typhus fever, combined with congestion, if not with inflammation, in the viscera. One class of reasoners, zealous for the vindication of cow-pox from every reflection, will therefore say, that this was a singular case, in which death ought not to be imputed to the small-pox, but to other accidental circumstances in the natural constitution of the patient, or in the form of the disease; while their antagonists will reject such an explanation, because every bad case of small-pox might be accounted for in the same manner with equal plausibility. In whatever light different theorists may consider it, I feel fully assured, that such occurrences will ever be extremely rare,—that other circumstances besides the fatal issue will declare their nature to be peculiar, and that they will not, on the whole, detract from the utility of vaccination."

The following are the general inferences drawn by the author.

"I found (says he,) no particular reason for disputing the genuineness of the vaccine disease which the fifty-four patients had received by inoculation. Ten of them were vaccinated by medical practitioners. This was comparatively a small number. But I could find no materials from which a judgment could be formed of the proportion which the persons attacked bore to the total number of operations performed under medical inspection, and a comparison made with the proportion of attacks among others. It is probable that a small proportion of the inoculations in the place were performed by medical men. The facility and certainty of the vaccine operation in any person's hands, were, at its first introduction, strongly inculcated among its recommendations. I could not learn that any record of vaccinations had been kept."

This last circumstance is very properly regretted by Dr. Dewar. At the same time it must be remarked, that the general uniformity of the variolous symptoms might lead us to suspect that the previous vaccination was similar. The author still entertains the same favourable opinion of vaccination, and conceives that the notions concerning spurious pustules are unfounded. His arguments on this subject seem reasonable.

"If (says he) the generality of the Cupar vaccinations were spurious, we must pronounce the whole vaccine disease, as known in that part of the country, to be spurious: we must conclude that there is an important but refined distinction on the subject which cannot be conveyed by books, nor transmitted from one person to another, (even with all the phenomena before them,) to any considerable distance of succession. This would evidently be in itself a material detraction from the value of vaccination. No such perplexing anomalies occurred in the variolous inoculation."

All this is very just; yet we cannot help contrasting these numerous cases with the uniform success which, by the reports from the Small-Pox Hospital, have attended the practice of that establishment. The test proposed by Mr. Brice, and recommended by Dr. Dewar, has rarely been applied at the above place; and it is known, that, after the test in other places, the mitigated small-pox has occurred.

All the conclusions of the author are in favour of vaccination on the above plan. An exact register of every case also is particularly recommended.

---

*An Account of some Experiments made with the Vapour of Boiling Tar, in the Cure of Pulmonary Consumption;* by Alexander Crichton, M.D. F.R.S. Physician in ordinary to their Imperial Majesties the Emperor and Dowager-Empress of Russia, Physician-in-chief of the Civil Department of the Empire, Knight of the Order of St. Valdimir, Honorary Member of the Imperial Academy of Sciences of St. Petersburgh, Fellow of the Royal Linnæan Society of London, Corresponding Member of the Royal Society of Gottingen, and Member of various other learned Societies. 8vo. pp. 62. Underwood, London.

It will be enough to give the inferences drawn from the cases.

It must be evident (says Dr. Crichton) from the preceding cases, that the tar fumigation, though completely successful in some of them, did not produce the same good in all; but, on the other hand, the very great relief which every patient experienced at first from it, particularly in the diminution of cough, expectoration, and hectic fever, is a fact which ought to encourage us to multiply the trials of this remedy as far as possible.

"There is an evident cause for its want of complete success in the hospital, and which demonstrates the necessity of having a building constructed purposely for the treatment of consumptive patients,—I mean, the want of room obliging us to place several of these patients together in one ward. Thus, the malady is kept up by the infection of their breaths, this cause constantly acting on their lungs as a real contagion. Every physician knows the power which the breath of a consumptive person has in producing this disease in others, even in such as have no apparent disposition to consumption."

Notwithstanding all this evidence, we confess ourselves in a state of scepticism. It is no new thing to propose inhalations, and even balsamic inhalations. Dr. Fothergill some time ago remarked the inefficacy of these substances taken by the mouth, and even doubted if they were not injurious. After this, a gentleman, who called himself Dr. Sterne, adventured a chemical preparation, by which a balsamic substance might be inhaled into the lungs; thus finding a direct passage, and inducing no stimulus on the digestive organs. Many of the faculty found the plan advantageous; and we doubt not that such is the case with the tar fumigations. But, after long and close examination and inquiry, we have never been able to detect the infectious property of consumption, and are rather disposed to impute the opinion to the frequency with which it affects a number of brothers and sisters from the same predisposing cause. In most of these instances, however, the mother who nurses them all survives them all.

"The patients (continues Dr. C.) who have derived the greatest benefit from the tar-vapour, are those who were attacked with true scrofulous, or tubercular, phthisis. I confess this is quite contrary to what I expected. This kind of phthisis is the most common in Russia, as in all northern climates.

"The tar-vapour seems to have healed the ulcers, and removed the inflammation of the tubercles in the greater number of such cases; but, I do not believe it produces the absorption of the tubercles themselves. My first patient, N., although well enough to attend to his business out of doors, and, although he has regained his strength, has still some symptoms, which, to the eye of an observing physician, announce the presence of these tumours in his lungs; but, so long as they remain inactive, that is to say, without inflammation or ulceration, life may be prolonged: and, if circumstances allowed this patient to reside in a better climate, there is every probability that he might live long.

"In cases of phthisis, derived from a large abscess or vomica, particularly in young persons of sanguineous constitutions, where suppuration goes on rapidly, and is generally accompanied by fever, the tar-vapour does little good. The immense quantity of purulent matter constantly present in the cavities of these large abscesses, prevents the vapour from acting upon their surface, and consequently it loses its effect. In two cases of this kind, a very temporary relief only took place from the fumigation. Indeed, it must be in vain to expect any great success from this, or from any other remedy, in cases of violent and neglected inflammation of the lungs, terminating in large abscesses, the disorganization of the lungs in these cases being too widely extended to admit of cure.

"Nearly the same thing may be said of cases of suppuration succeeding active hæmorrhages in young persons, especially when accompanied with fever.

"In one case, however, of this kind, I succeeded in removing the consumptive symptoms by the tar-fumigation, and the use of acetate of lead, combined with the aqueous extract of opium, which the patient continued to take during a whole month, without experiencing cholic, or any other inconvenience."

Here again we are at issue with the author. We have often found patients recover after considerable abscesses, if the season and other circumstances have been favourable: but, with tubercles, we have fancied our success less. The constitutional disposition to form them continuing, it commonly happens that a succession of them continue to suppurate and open into the air-cells, whilst new ones are forming. Hence, though the patient has frequent respites, which all but the medical people impute to remedies, the disease is gradually destroying more of the lungs. But, in cases of large abscess from suppuration after active inflammation, the rest of the lungs is often sound, and the patient recovers, if the season and other events are favourable.

### MEDICAL AND PHILOSOPHICAL INTELLIGENCE.

*To the Editors of the London Medical and Physical Journal.*

GENTLEMEN,

